# Socioeconomic Predictors of Trends in Cancer Mortality among Municipalities in Japan, 2010–2019

**DOI:** 10.31557/APJCP.2021.22.2.499

**Published:** 2021-02

**Authors:** Tasuku Okui

**Affiliations:** *Medical Information Center, Kyushu University Hospital, Fukuoka City, Japan. *

**Keywords:** Cancer, mortality, municipalities, urbanization, vital statistics

## Abstract

**Background:**

A study investigating associations between various socioeconomic factors and standardized mortality ratios (SMR) of each type of cancer among municipalities in Japan has not been conducted using the data of the past decade. Herein, we investigated the predictors of a recent trend of municipal SMRs of cancer using the Vital Statistics in Japan and revealed the change in the SMRs depending on the identified predictors.

**Methods:**

Data on cancer mortality for each municipality in 2010 and 2019 were used. We calculated empirical Bayes SMR (EBSMR) for each municipality by type of cancer and sex and then fitted a multiple linear regression model using possible predictors in 2010 as explanatory variables and the EBSMR in 2019 as the outcome variable. We also classified municipalities into quintiles based on the values of an identified predictor in 2010, and SMRs of each type of cancer in 2010 and 2019 were calculated for each quintile.

**Results:**

The total population was positively associated with EMSMRs of multiple cancer types, whereas educational level was negatively associated with EMSMRs of multiple cancer types. In addition, SMRs of municipalities with the lowest educational level deteriorated from 2010 to 2019 for many cancer types among men and women, and the difference between municipalities with the highest and lowest educational level for the SMR of cancer in all sites widened in 2019 for men. On the other hand, the SMR of municipalities with the highest educational level or the largest total population tended to be higher than municipalities with lower counterparts in both 2010 and 2019 for women.

**Conclusion:**

There was a difference in the trend of the SMRs of multiple types of cancer depending on municipal educational level, whereas municipalities with larger population or educational level continued to have higher SMRs of cancer in all sites for women.

## Introduction

Cancer is the leading cause of death in Japan, and the mortality rate has increased over time along with the aging of population (Ministry of Health, Labour and Welfare of Japan, 2020). In contrast, age standardized mortality rate (SMR) has decreased in recent years for many types of cancer (Ministry of Health, Labour and Welfare of Japan, 2020). However, it is known that cancer mortality and incidence rates vary depending on the socioeconomic status (SES) of individuals and regions (Yost et al., 2001; Fukuda et al., 2005). Socioeconomic disparities in health has become a major theme in Japanese public health in recent years (Kagamimori et al., 2009), and it has been demonstrated that multiple types of health behaviors and disease prevalence are associated with SES in Japan (Fukuda, et al., 2005; Fujita, et al., 2016). Regarding cancer, some epidemiological studies have indicated socioeconomic disparities in the cancer survival rate in Japan. A difference has been reported in gastric cancer survival among occupational classes (Kuwahara et al., 2010). In addition, SES differences in cervical and corpus cancer survival have been reported in another study (Ueda et al., 2006).

The cancer mortality rate also varies depending on regional SES. The difference in SMR of cancer in all sites between metropolitan and non-metropolitan areas has been shown in the United States (Singh et al., 2014). Regarding the association between municipal cancer mortality and regional SES in Japan, although some studies have investigated the association using the Vital Statistics in Japan, the results of an association between cancer mortality rate and regional SES vary depending on previous studies in Japan. According to a previous study investigating the association between municipal SES and SMRs of representative types of cancer using the data of the period from 1993 to 1998 in Japan (Fukuda et al., 2005), the SMRs were shown to be rather higher in municipalities with higher SES. In addition, according to a study investigating the SMRs of cancer in all sites for the 10 largest Japanese cities using the data of the period from 2003 to 2007 (Kano et al., 2013), the SMRs for the 10 largest Japanese cities tended to be larger than all of Japan. On the other hand, according to a study investigating the association between geographic deprivation level (poverty level) and SMRs of cancer using the data of the period from 2003 to 2007 (Nakaya et al., 2011), municipalities with a high poverty level tended to have higher mortality rate for cancer in all sites. The association between SES and municipal SMR of cancer is believed to vary depending on the definition of SES, and the association may also change depending on time. However, a study investigating associations between multiple kinds of socioeconomic factors and SMRs of each type of cancer among municipalities in Japan has not been conducted using the data of the past decade. It is important to investigate which socioeconomic factor is positively or negatively associated with the recent trend of municipal SMRs of cancer to discuss the preventive method of increase of SMR of a municipality.

In this study, we investigated the predictors of recent trend of municipal SMRs of cancer using the Vital Statistics in Japan and revealed the change in the municipal SMRs depending on the identified predictors.

## Materials and Methods

*Data*

The Vital Statistics data of 2010 and 2019 were used for cancer mortality data in this study (Ministry of Health, Labour and Welfare of Japan, 2020), and the data of cancer mortality for each municipality and sex were used. Regarding type of cancers, we evaluated cancer in all sites, stomach cancer, colorectal cancer, liver cancer, gallbladder cancer, pancreatic cancer, lung cancer, and breast cancer. The International Classification of Diseases (10th Revision) codes for each type of cancer is as follows: cancer in all sites (C00–97), stomach cancer (C16), colorectal (C18–20), liver (C22), gallbladder (C23–24), pancreas (C25), lung (C33–34), and breast (C50) (Ministry of Health, Labour and Welfare of Japan, 2020). The population data of each municipality for each age group and sex were obtained from the national survey of population, demographics, and households using the basic resident register (Ministry of Internal Affairs and Communications, 2020). Data for age groups in 5-year increments from 0 to 4 through 75 to 79 years and individuals older than 80 years were used for analysis. Because we focused on the changes in SMRs from 2010 to 2019 in the analysis, municipalities that existed in both 2010 and 2019 were used in the analysis. Therefore, municipalities that were integrated by 2019 were not used in the analysis, and municipalities for which only the names were changed before 2019 were used in the analysis.

As possible predictors of cancer mortality, we used the data of number of births, single households, elderly households, fatherless households, marriages, divorces, unemployed individuals, labor workers, farmers, self-employed individuals, university graduates, hospitals, physicians, and hospital beds for each municipality. The data of number of births, marriages, and divorces were extracted from the Vital Statistics (Ministry of Internal Affairs and Communications, 2020), and the data of number of single households, elderly households, fatherless households, unemployed individuals, labor workers, farmers, self-employed individuals, and university graduates were extracted from the Census (Ministry of Internal Affairs and Communications, 2020). In addition, number of hospitals and hospital beds were extracted from the Survey of Medical Institutions (Ministry of Internal Affairs and Communications, 2020), and number of physicians was extracted from the Survey of Physicians, Dentists and Pharmacists (Ministry of Internal Affairs and Communications, 2020). Educational level was defined as number of university graduates per 1,000 persons over 25 years old. The data of population, taxable income, financial capability index were also used. The data of taxable income were extracted from the Survey of taxation status of municipal tax (Ministry of Internal Affairs and Communications, 2020), and those of the financial capability index were extracted from the Ministry of Internal Affairs and Communications (Ministry of Internal Affairs and Communications, 2020). The financial capability index can be calculated by dividing standard financial revenues with the amount of basic fiscal demand and indicates the financial ability of a municipality (Ando, 2017).

*Statistical analysis*

SMR is the ratio of the observed mortality rate in a region to the expected mortality rate of that region (Leyland et al., 2005), and it is the mortality rate ratio taking into account the difference in the age composition of the regions (Leyland et al., 2005; Taylor, 2013). First, we calculated the expected mortality for each municipality using age group-specific population data and the age group-specific mortality rate for all of Japan by type of cancer and sex. From the expected number of cancer and actual mortality, we calculated empirical Bayes SMRs (EBSMR) for each municipality by type of cancer and sex using DCluster (https://cran.r-project.org/web/packages/DCluster/DCluster.pdf). EBSMR is often calculated when there are regions in which the population is small and the SMRs become unreliable (Leyland et al., 2005), and the only difference between SMR and EBSMR is the calculation method. A regression model with a prior distribution is used in the calculation of EBSMRs, and the SMRs (EBSMRs) of regions with small populations are estimated by borrowing information from the prior distribution whose parameters are empirically estimated from the data (Leyland et al., 2005). We fitted a multiple linear regression model using possible predictors in 2010 as explanatory variables and EBSMR in 2019 as the outcome variable for each type of cancer by sex. By using the data of multiple time points for the regression analysis and including EBSMR in 2010 as an explanatory variable, we could infer which predictor affected the trend in EBSMRs of municipalities from 2010 to 2019.

We also classified municipalities into quintiles based on the values of an identified predictor in 2010. SMRs of each type of cancer were calculated for each quintile in 2010 and 2019. Based on this analysis, we could assess the difference in the SMRs based on the values of the predictors and could verify how the SMRs of municipalities changed depending on the identified predictors. All statistical analyses were conducted using R 3.6.3 software (https://www.r-project.org/).

## Results

Table 1 details the basic characteristics of municipalities used in the analysis. The data of 1739 municipalities in total were used in the analysis.

Table 2 shows the results of multiple linear regression analysis for each type of cancer among men. The population, number of single households, and the EBSMR in 2010 were positively associated with the EMSMR in 2019 for multiple types of cancer. On the other hand, number of elderly households and educational level were negatively associated with the outcomes in many cases.

Table 3 demonstrates the results of multiple linear regression analysis for each type of cancer among women. Predictors that were associated with the outcomes were relatively similar to those in men, whereas a significant negative association was observed for population in the results of stomach and gallbladder cancer.

Because standardized partial regression coefficients of population and educational level were particularly high from the results of Tables 2 and 3, we classified municipalities into quintiles based on the values of each of the two predictors. In addition, we calculated SMRs for each quintile in 2010 and 2019 by sex and type of cancer. The population and educational level for each of municipal quintiles are shown in the supplementary information.

**Figure 1:**
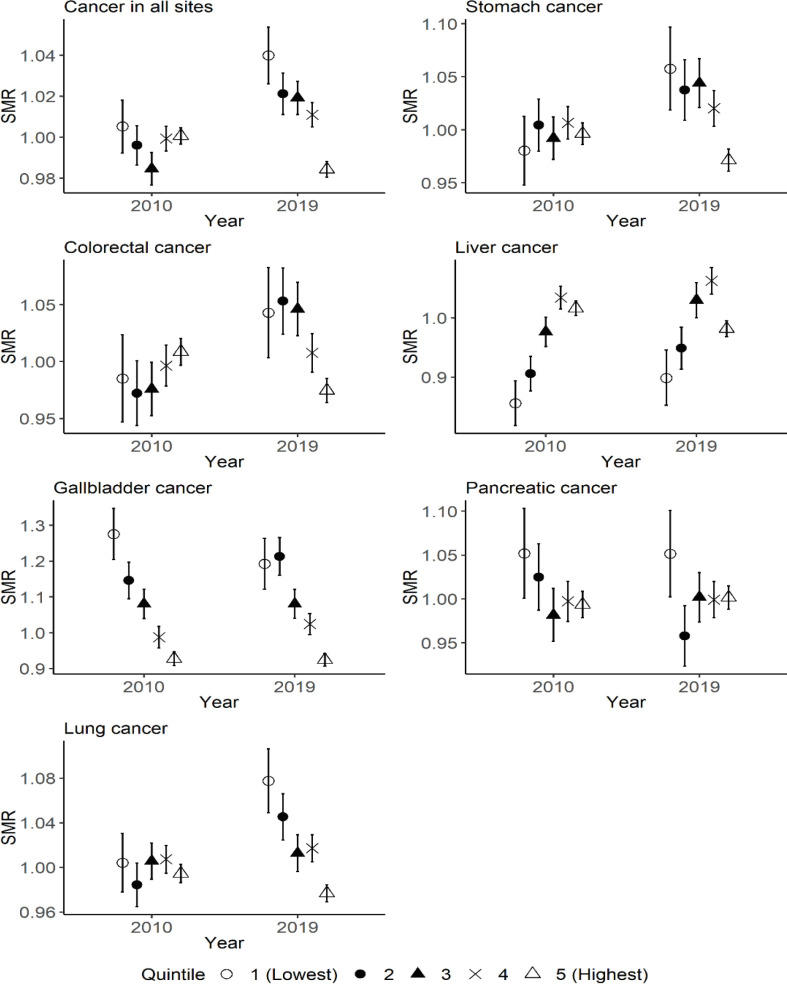
The Difference in the SMRs for Each Type of Cancer among Quintiles of Municipal Educational Level in 2010 and 2019 for Men. Quintile 1 corresponds to municipalities with the lowest educational level in 2010, and quintile 5 corresponds to municipalities with the highest educational level in 2010. The range of error bar indicates the 95% confidence interval of SMR for each quintile

Figure 1 shows the difference in the SMRs among quintiles of municipal educational level in 2010 and 2019 for each type of cancer in men. The relationships between educational level and SMRs were different depending on type of cancer. Although the SMR was already the highest in the quintile with the lowest educational level for cancer in all sites in 2010, the SMR deteriorated further in 2019. A similar surge of SMR in the quintile with the lowest educational level was observed for stomach, colorectal, and lung cancers. The SMRs of municipalities with the highest educational level ameliorated in 2010 to 2019 for many of the types of cancers. The relationship between municipalities with the highest and lowest educational levels for the SMR of cancer in all sites widened in 2019.

**Figure 2:**
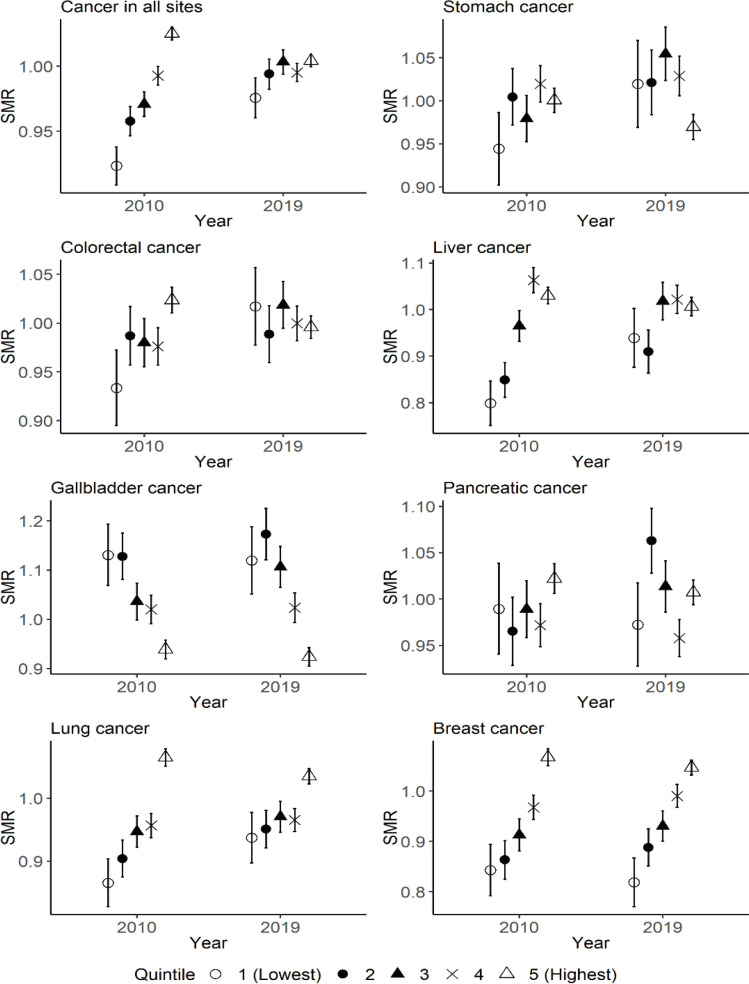
The Difference in the SMRs for Each Type of Cancer among Quintiles of Municipal Educational Level in 2010 and 2019 for Women. Quintile 1 corresponds to municipalities with the lowest educational level in 2010, and quintile 5 corresponds to municipalities with the highest educational level in 2010. The range of error bar indicates the 95% confidence interval of SMR for each quintile

Figure 2 demonstrates the difference in the SMRs among quintiles of municipal educational level in 2010 and 2019 for each type of cancer in women. The SMRs tended to be high in quintiles with higher educational level for multiple types of cancer in 2010, whereas the difference in the SMRs of cancer in all sites between municipalities with the highest and lowest educational levels decreased from 2010 to 2019.

**Figure 3:**
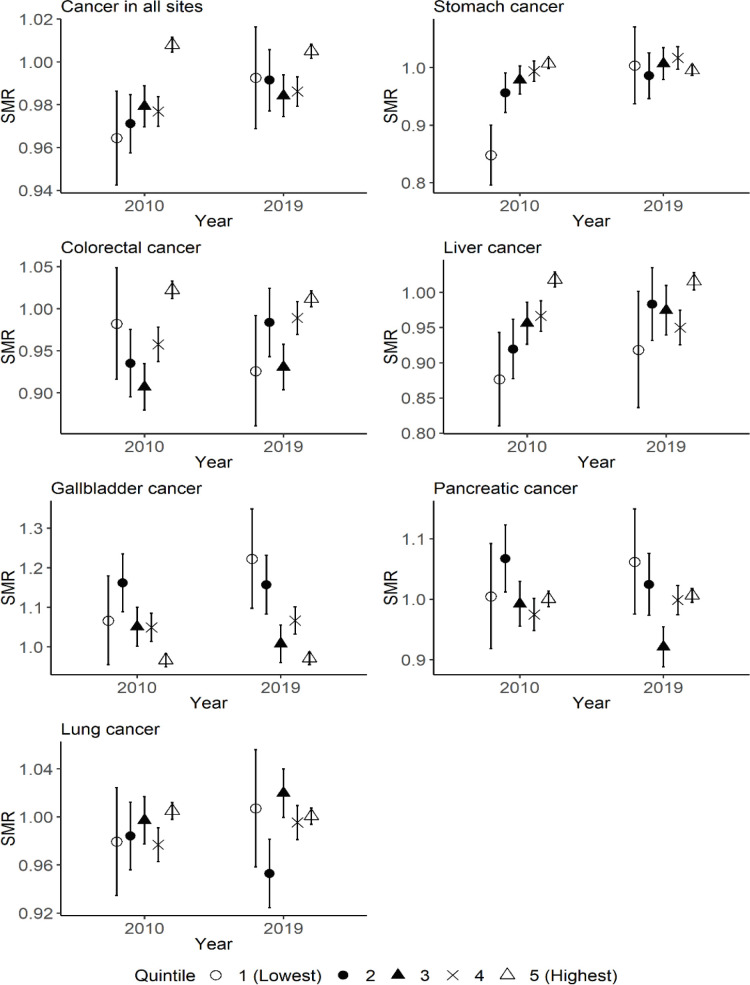
The Difference in the SMRs for Each Type of Cancer among Quintiles of Municipal Population in 2010 and 2019 for Men. Quintile 1 corresponds to municipalities with the lowest level of total population in 2010, and quintile 5 corresponds to municipalities with the highest level of total population in 2010. The range of error bar indicates the 95% confidence interval of SMR for each quintile

Figure 3 indicates the difference in the SMRs among quintiles of municipal population in 2010 and 2019 for each type of cancer in men. Although the SMR of cancer in all sites in the quintile with the highest financial capability was significantly higher than the other quintiles in 2010, the difference among quintiles decreased in 2019.

**Figure 4:**
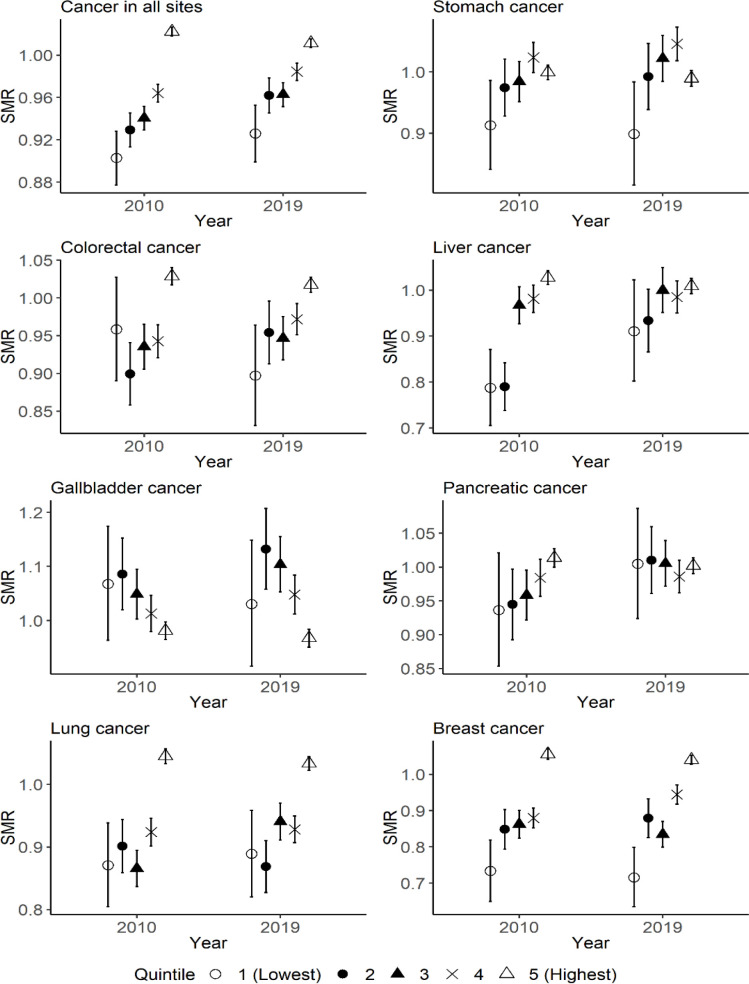
The Difference in the SMRs for Each Type of Cancer among Quintiles of Municipal Population in 2010 and 2019 for Women. Quintile 1 corresponds to municipalities with the lowest level of total population in 2010, and quintile 5 corresponds to municipalities with the highest level of total population in 2010. The range of error bar indicates the 95% confidence interval of SMR for each quintile

Figure 4 indicates the difference in the SMRs among quintiles of municipal population in 2010 and 2019 for each type of cancer in women. The SMRs in the quintile with the highest financial capability was significantly higher than the other quintiles in both 2010 and 2019 for cancer in all sites. Similar trends were observed for colorectal, lung, and breast cancers.

**Table 1:** Basic Characteristics of Municipalities Used in the Analysis

Characteristics	2010 (N = 1739)	2019 (N = 1739)
Population	26014.0 (9180.0-65848.5)	
Number of births*	38.4 (33.4-43.6)	
Number of single households†	251.5 (204.5-301.8)	
Number of elderly households†	213.4 (172.9-274.3)	
Number of fatherless households†	13.7 (10.6-17.0)	
Number of marriages‡	426.8 (351.3-519.7)	
Number of divorces‡	173.2 (138.8-206.7)	
Number of unemployed persons§	61.2 (50.6-73.1)	
Number of labor workers§	66.7 (59.2-77.5)	
Number of farmers§	71.8 (25.2-152.8)	
Number of self-employed persons§	167.9 (118.8-238.3)	
Financial capability index	0.47 (0.27-0.73)	
Taxable income per capita||	1050.4 (866.5-1249.5)	
Educational level¶	106.3 (76.8-154.7)	
Number of hospitals‡	6.1 (2.0-10.0)	
Number of physicians‡	124.2 (71.3-183.0)	
Number of hospital beds‡	1037.2 (434.1-1743.3)	
Mortality for men*		
Cancer in all sites	383.8 (310.8-475.8)	405.0 (332.6-502.8)
Stomach cancer	55.7 (39.9-77.2)	48.7 (34.0-68.4)
Colorectal cancer	38.7 (26.3-55.1)	45.8 (31.8-62.8)
Liver cancer	32.7 (20.0-50.1)	27.0 (15.7-40.5)
Gallbladder cancer	13.1 (4.2-24.0)	14.9 (5.8-25.8)
Pancreatic cancer	23.7 (14.4-35.9)	29.8 (18.6-43.3)
Lung cancer	89.0 (65.7-118.5)	94.3 (69.7-126.1)
Mortality for women*		
Cancer in all sites	237.6 (194.7-290.7)	264.7 (216.9-330.7)
Stomach cancer	27.5 (17.0-41.2)	23.6 (13.6-36.2)
Colorectal cancer	31.3 (21.0-46.1)	37.6 (25.3-55.1)
Liver cancer	15.3 (5.1-24.1)	11.6 (0.0-20.7)
Gallbladder cancer	14.0 (6.4-25.0)	12.9 (4.5-23.6)
Pancreatic cancer	20.3 (12.0-31.2)	29.1 (19.2-43.7)
Lung cancer	28.8 (18.7-41.8)	34.0 (22.4-48.2)
Breast cancer	16.7 (6.3-24.2)	20.6 (9.7-29.0)

Each value indicates median (interquartile range). The values of the upper part of the characteristic in 2019 are not shown because they were not used in the analysis; *, Number per 1,000 women in 15-49 years old; †, Number per 1,000 households; ‡, Number per 100,000 persons; §, Number per 1,000 labor force persons; ||Unit, 1,000 yen; ¶Number of university graduates per 1,000 persons over 25 years old.

**Table 2:** Result of Multiple Linear Regression Analysis for Eeach Type of Cancer among Men

Variables	Cancer in all sites	Stomach cancer	Colorectal cancer	Liver cancer
	SPRC (95% CI)	SPRC (95% CI)	SPRC (95% CI)	SPRC (95% CI)
Population	0.09 (0.04, 0.14)	-0.04 (-0.09, 0.01)	0.07 (0.02, 0.12)	0.07 (0.02, 0.11)
Number of births*	-0.07 (-0.12, -0.01)	-0.06 (-0.12, -0.00)	-0.06 (-0.12, -0.01)	-0.02 (-0.07, 0.04)
Number of single households†	0.16 (0.10, 0.22)	0.04 (-0.03, 0.11)	0.15 (0.08, 0.22)	0.06 (-0.01, 0.12)
Number of elderly households†	-0.15 (-0.22, -0.08)	-0.10 (-0.17, -0.03)	-0.16 (-0.23, -0.09)	0.01 (-0.06, 0.08)
Number of fatherless households†	0.08 (0.02, 0.14)	0.01 (-0.06, 0.07)	0.04 (-0.02, 0.10)	0.09 (0.03, 0.15)
Number of marriages‡	-0.01 (-0.08, 0.06)	-0.05 (-0.13, 0.03)	0.02 (-0.06, 0.09)	0.04 (-0.03, 0.12)
Number of divorces‡	0.05 (-0.01, 0.10)	0.01 (-0.04, 0.07)	0.03 (-0.02, 0.09)	0.07 (0.02, 0.13)
Number of unemployed persons§	0.04 (-0.02, 0.10)	-0.02 (-0.08, 0.04)	0.03 (-0.04, 0.09)	-0.01 (-0.07, 0.05)
Number of labor workers§	0.03 (-0.02, 0.08)	0.03 (-0.03, 0.08)	0.01 (-0.05, 0.06)	0.00 (-0.05, 0.05)
Number of farmers§	-0.05 (-0.15, 0.06)	-0.13 (-0.24, -0.02)	-0.07 (-0.19, 0.04)	-0.07 (-0.17, 0.04)
Number of self-employed persons§	-0.00 (-0.11, 0.10)	0.04 (-0.07, 0.15)	0.00 (-0.11, 0.12)	0.05 (-0.06, 0.16)
Financial capability index	-0.04 (-0.11, 0.03)	0.04 (-0.04, 0.11)	-0.04 (-0.12, 0.03)	0.01 (-0.06, 0.08)
Taxable income per capita||	-0.02 (-0.12, 0.07)	-0.11 (-0.21, -0.01)	0.01 (-0.10, 0.11)	-0.09 (-0.19, 0.01)
Educational level¶	-0.30 (-0.39, -0.22)	-0.10 (-0.19, -0.00)	-0.29 (-0.38, -0.19)	-0.06 (-0.15, 0.03)
Number of hospitals‡	-0.04 (-0.10, 0.03)	-0.01 (-0.08, 0.06)	-0.04 (-0.10, 0.03)	0.03 (-0.04, 0.09)
Number of physicians‡	-0.01 (-0.06, 0.05)	0.00 (-0.06, 0.06)	-0.03 (-0.09, 0.03)	-0.02 (-0.07, 0.04)
Number of hospital beds‡	-0.00 (-0.07, 0.07)	-0.04 (-0.11, 0.04)	0.02 (-0.05, 0.09)	0.01 (-0.06, 0.09)
EBSMR of 2010	0.30 (0.25, 0.34)	0.30 (0.26, 0.35)	0.19 (0.14, 0.24)	0.35 (0.31, 0.40)
Population	-0.05 (-0.10, 0.00)	0.09 (0.03, 0.14)	0.08 (0.03, 0.13)	
Number of births*	-0.00 (-0.06, 0.05)	-0.01 (-0.07, 0.05)	-0.04 (-0.10, 0.01)	
Number of single households†	0.05 (-0.02, 0.12)	0.01 (-0.06, 0.08)	0.08 (0.02, 0.15)	
Number of elderly households†	-0.08 (-0.15, -0.00)	-0.00 (-0.08, 0.07)	-0.08 (-0.15, -0.01)	
Number of fatherless households†	-0.01 (-0.08, 0.05)	-0.00 (-0.07, 0.06)	0.09 (0.03, 0.15)	
Number of marriages‡	-0.01 (-0.09, 0.07)	0.01 (-0.07, 0.09)	-0.00 (-0.08, 0.07)	
Number of divorces‡	-0.02 (-0.08, 0.04)	-0.01 (-0.07, 0.05)	0.03 (-0.02, 0.09)	
Number of unemployed persons§	0.03 (-0.03, 0.10)	-0.01 (-0.08, 0.05)	0.00 (-0.06, 0.06)	
Number of labor workers§	0.01 (-0.05, 0.07)	0.01 (-0.04, 0.07)	0.01 (-0.04, 0.07)	
Number of farmers§	0.06 (-0.05, 0.18)	0.02 (-0.10, 0.13)	-0.08 (-0.19, 0.03)	
Number of self-employed persons§	-0.01 (-0.13, 0.11)	-0.07 (-0.19, 0.04)	0.02 (-0.09, 0.13)	
Financial capability index	-0.01 (-0.09, 0.07)	-0.09 (-0.17, -0.01)	-0.02 (-0.10, 0.05)	
Taxable income per capita||	0.08 (-0.03, 0.18)	-0.02 (-0.13, 0.09)	0.00 (-0.10, 0.10)	
Educational level¶	-0.21 (-0.30, -0.11)	-0.01 (-0.10, 0.09)	-0.28 (-0.37, -0.19)	
Number of hospitals‡	-0.05 (-0.11, 0.02)	-0.00 (-0.07, 0.07)	-0.05 (-0.11, 0.02)	
Number of physicians‡	0.00 (-0.06, 0.07)	-0.02 (-0.08, 0.04)	0.01 (-0.04, 0.07)	
Number of hospital beds‡	0.05 (-0.03, 0.12)	0.02 (-0.06, 0.09)	0.01 (-0.06, 0.08)	
EBSMR of 2010	0.07 (0.02, 0.12)	0.03 (-0.02, 0.08)	0.26 (0.21, 0.30)	

EBSMR, Empirical Bayes standardized mortality ratio; SPRC, Standardized partial regression coefficient; CI, Confidence interval; *, Number per 1,000 women in 15-49 years old; †, Number per 1,000 households; ‡, Number per 100,000 persons; §, Number per 1,000 labor force persons; ||Unit, 1,000 yen; ¶, Number of university graduates per 1,000 persons over 25 years old

**Table 3:** Result of Multiple Linear Regression Analysis for Each Type of Cancer among Women

Variables	Cancer in all sites	Stomach cancer	Colorectal cancer	Liver cancer
	SPRC (95% CI)	SPRC (95% CI)	SPRC (95% CI)	SPRC (95% CI)
Population	0.12 (0.07, 0.17)	-0.10 (-0.15, -0.05)	0.09 (0.04, 0.14)	0.03 (-0.02, 0.08)
Number of births*	-0.06 (-0.11, -0.00)	-0.00 (-0.06, 0.06)	-0.02 (-0.08, 0.04)	-0.01 (-0.06, 0.05)
Number of single households†	0.11 (0.04, 0.18)	0.01 (-0.06, 0.08)	0.05 (-0.02, 0.12)	0.02 (-0.04, 0.09)
Number of elderly households†	-0.11 (-0.18, -0.04)	-0.09 (-0.16, -0.01)	-0.08 (-0.15, -0.00)	0.03 (-0.04, 0.10)
Number of fatherless households†	0.05 (-0.01, 0.11)	0.00 (-0.06, 0.07)	0.05 (-0.01, 0.12)	0.06 (-0.01, 0.12)
Number of marriages‡	-0.00 (-0.08, 0.08)	-0.10 (-0.18, -0.03)	-0.07 (-0.15, 0.01)	0.02 (-0.06, 0.09)
Number of divorces‡	0.04 (-0.01, 0.10)	-0.01 (-0.07, 0.05)	-0.02 (-0.08, 0.04)	0.05 (-0.01, 0.10)
Number of unemployed persons§	0.02 (-0.05, 0.08)	-0.02 (-0.09, 0.04)	-0.02 (-0.08, 0.05)	0.05 (-0.02, 0.11)
Number of labor workers§	0.01 (-0.04, 0.06)	-0.00 (-0.06, 0.05)	0.02 (-0.04, 0.07)	0.02 (-0.03, 0.07)
Number of farmers§	0.00 (-0.11, 0.12)	-0.09 (-0.20, 0.03)	-0.05 (-0.17, 0.06)	-0.05 (-0.17, 0.06)
Number of self-employed persons§	-0.10 (-0.21, 0.01)	0.05 (-0.06, 0.16)	-0.02 (-0.14, 0.09)	0.08 (-0.03, 0.19)
Financial capability index	-0.05 (-0.12, 0.02)	0.10 (0.02, 0.17)	0.01 (-0.07, 0.08)	0.06 (-0.02, 0.13)
Taxable income per capita||	0.08 (-0.02, 0.18)	-0.00 (-0.11, 0.10)	0.07 (-0.03, 0.18)	-0.06 (-0.16, 0.04)
Number of university graduates¶	-0.23 (-0.32, -0.14)	-0.10 (-0.19, -0.00)	-0.19 (-0.29, -0.10)	-0.02 (-0.12, 0.07)
Number of hospitals‡	-0.05 (-0.11, 0.02)	-0.04 (-0.10, 0.03)	-0.08 (-0.15, -0.01)	0.03 (-0.04, 0.09)
Number of physicians‡	-0.05 (-0.11, 0.01)	-0.01 (-0.07, 0.05)	-0.03 (-0.09, 0.03)	-0.01 (-0.07, 0.04)
Number of hospital beds‡	0.06 (-0.02, 0.13)	-0.01 (-0.08, 0.07)	0.07 (-0.01, 0.14)	0.02 (-0.06, 0.09)
EBSMR of 2010	0.23 (0.18, 0.28)	0.18 (0.14, 0.23)	0.15 (0.11, 0.20)	0.28 (0.24, 0.33)
Population	-0.11 (-0.16, -0.06)	-0.01 (-0.06, 0.04)	0.14 (0.09, 0.19)	0.26 (0.21, 0.31)
Number of births*	0.00 (-0.05, 0.06)	-0.05 (-0.11, 0.01)	-0.10 (-0.16, -0.05)	-0.04 (-0.10, 0.01)
Number of single households†	-0.00 (-0.07, 0.07)	0.04 (-0.03, 0.11)	0.11 (0.04, 0.17)	0.04 (-0.02, 0.11)
Number of elderly households†	-0.04 (-0.12, 0.03)	-0.05 (-0.13, 0.02)	-0.02 (-0.09, 0.05)	-0.01 (-0.08, 0.06)
Number of fatherless households†	0.04 (-0.03, 0.10)	0.02 (-0.05, 0.09)	0.04 (-0.02, 0.10)	0.00 (-0.06, 0.07)
Number of marriages‡	0.00 (-0.08, 0.08)	0.02 (-0.06, 0.10)	0.08 (0.01, 0.16)	0.01 (-0.07, 0.08)
Number of divorces‡	-0.02 (-0.08, 0.04)	0.01 (-0.05, 0.07)	0.09 (0.04, 0.15)	0.02 (-0.03, 0.08)
Number of unemployed persons§	-0.05 (-0.11, 0.02)	0.01 (-0.06, 0.07)	0.02 (-0.04, 0.08)	0.02 (-0.04, 0.08)
Number of labor workers§	-0.01 (-0.06, 0.05)	-0.01 (-0.06, 0.05)	0.02 (-0.03, 0.07)	0.00 (-0.05, 0.06)
Number of farmers§	-0.07 (-0.18, 0.05)	0.06 (-0.06, 0.18)	0.04 (-0.07, 0.15)	0.06 (-0.05, 0.17)
Number of self-employed persons§	-0.01 (-0.12, 0.11)	-0.08 (-0.20, 0.04)	-0.08 (-0.18, 0.03)	-0.08 (-0.19, 0.03)
Financial capability index	0.01 (-0.06, 0.09)	-0.12 (-0.20, -0.04)	-0.03 (-0.10, 0.04)	-0.08 (-0.15, -0.01)
Taxable income per capita||	-0.05 (-0.16, 0.05)	0.05 (-0.06, 0.16)	0.01 (-0.09, 0.11)	0.03 (-0.07, 0.13)
Educational level¶	-0.19 (-0.29, -0.09)	-0.01 (-0.11, 0.08)	-0.09 (-0.18, -0.00)	0.08 (-0.01, 0.18)
Number of hospitals‡	-0.01 (-0.08, 0.06)	-0.03 (-0.10, 0.04)	-0.01 (-0.07, 0.06)	-0.01 (-0.08, 0.06)
Number of physicians‡	0.04 (-0.02, 0.10)	-0.01 (-0.08, 0.05)	-0.03 (-0.09, 0.03)	-0.02 (-0.08, 0.04)
Number of hospital beds‡	0.03 (-0.05, 0.10)	0.05 (-0.02, 0.13)	-0.00 (-0.07, 0.07)	0.00 (-0.07, 0.07)
EBSMR of 2010	0.06 (0.01, 0.11)	0.10 (0.05, 0.15)	0.23 (0.18, 0.28)	0.13 (0.08, 0.18)

EBSMR, Empirical Bayes standardized mortality ratio; SPRC, Standardized partial regression coefficient; CI, Confidence interval; *, Number per 1,000 women in 15-49 years old; †, Number per 1,000 households; ‡, Number per 100,000 persons; §, Number per 1,000 labor force persons; ||Unit, 1,000 yen; ¶, Number of university graduates per 1,000 persons over 25 years old.

## Discussion

The current study investigated predictors of trend of EBSMR of cancer using the Vital Statistics in Japan and revealed the trend of SMRs for municipalities based on identified predictors. We scrutinized possible reasons for the association for predictors that were shown to be associated with cancer in all sites.

Regarding the positive association between total population and EBSMRs for some types of cancer, it was evident that SMRs of colorectal, lung, and breast cancer were particularly high in the largest population quintile in both 2010 and 2019 for women. Urbanization has often been associated with higher cancer mortality rate in Japan. According to a study using the data of 1993 to 1998, clusters of colon and breast cancer tended to exist in urban-rich municipalities (Fukuda et al., 2005). Factors such as westernization of food and low birth rate were pointed out as possible reasons. In addition, the prevalence of smoking is known to be higher for women in urban areas of Japan (Fukuda et al., 2005), which might have contributed to the positive association for lung cancer. A positive association between urbanization and cancer incidence has also been reported in other countries (Momenyan et al., 2016; Chen et al., 2017), and higher incidence of breast and colorectal cancer in urban areas was also indicated in China (Li et al., 2018). Although population had a positive effect on EBSMRs in many of the types of cancer, the difference between SMRs of the highest and lowest population quintiles decreased in the period from 2010 to 2019 for some types of cancer. This is believed to be because population is associated with other factors of the municipalities. It is known that population of municipalities with higher educational level and taxable income level tended to be large in Japan (Fukuda et al., 2005).

The positive association with EBSMR was also observed for number of single households for some types of cancer. It is considered that an individual in a single household tends to be not married. Marital status is well known to be associated with the cancer mortality rate in Japan (Ikeda et al., 2007), with never-married, divorced, and widowed individuals shown to have higher cancer mortality rates. Social support obtained from partners is a factor for lower mortality rate in married individuals (Ikeda et al., 2007), and an individual in a single household does not receive support from cohabitants. In addition, living alone is also known to be associated with multiple unhealthy lifestyle behaviors (Bähler et al., 2016; Zhang et al., 2015), and smoking and drinking behavior is known to be associated with living arrangements. On the other hand, the number of elderly households was negatively associated with EBSMRs for some types of cancer. The number of elderly households was used as a factor composing geographic deprivation level of a municipality in a previous study in Japan (Nakaya et al., 2011), and the geographic deprivation level was shown to be associated with EBSMR of some types of cancer. However, this study suggested that the number of elderly households may have a positive effect on the municipal cancer mortality rate. Regarding the number of fatherless households, an association with EBSMRs was observed only in men, and the reason for the result is uncertain.

The educational level was also shown to be a predictor for cancer mortality. Educational level is known to be related to various types of health behaviors in Japan on an individual level (Tabuchi et al., 2017; Murakami et al., 2019). A strong association was observed particularly in colorectal, gallbladder, and lung cancers for men and women. Poor dietary habits, which are major risk factors for colorectal cancer, are known to be positively associated with low educational level in Japan (Nakamura et al., 2016). Smoking is a major risk factor for lung cancer, and a low educational level was shown to be associated with a higher smoking rate in Japan (Tabuchi et al., 2017). In contrast, taxable income was not shown to be a predictor, possibly because it is known that municipal educational level and taxable income correlates in Japan (Fukuda et al., 2005).

It was found that there was a difference in the trend of SMRs of some types of cancer depending on the educational level among municipalities. In the late 20th century, the SMR for cancer mortality rate was larger in municipalities with high socioeconomic positions in Japan (Fukuda et al., 2005). Dietary habits or lifestyle were also identified as factors. However, the association between municipal SES and SMR for cancer has changed in recent years, and municipalities with lower regional SES have been shown to have higher SMRs of cancer in all sites for men. To inhibit the current trend, regional disparities in educational level need to be addressed. On the other hand, SMRs tended to be high in quintiles with the largest population or the highest educational level for women, even in recent years. Therefore, an amelioration of lifestyle behaviors is needed for women living in urban-rich areas.

The current study has some limitations. Although there were cases in which even sign of coefficients of a predictor varied depending on type of cancer in the regression analysis, we cannot reveal the accurate reason for the association of a predictor and each type of cancer. The reason for the difference in the predictors depending on type of cancer need to be scrutinized in the future. There were also some mergers of municipalities during the analyzed periods, and there were cases in which the population of a municipality increased by merging with other municipalities. Moreover, we hypothesized the individuality of each municipality in the analysis, but spatial correlation might exist among adjacent municipalities. The analysis of data by a spatial data analysis method will be meaningful as the next step. Furthermore, this is an ecological study using regional data. In the future, a study using data of individual subjects is necessary to identify predictors while taking into account individual confounding factors.

In conclusion, this study investigated predictors of trends in EBSMR for each type of cancer using the Vital Statistics in Japan and revealed the trend of the SMRs based on the values of identified predictors from 2010 to 2019. The total population was positively associated with EMSMRs of multiple types of cancers, whereas a negative association was observed for educational level. It was also determined that SMRs of municipalities with the lowest educational level deteriorated in the period from 2010 to 2019 for many of the types of cancers among men and women, and the difference between municipalities with the highest and lowest financial capability widened in 2019 for cancer in all sites among men. On the other hand, the SMR of municipalities with the highest educational level tended to be higher than municipalities with lower counterparts in both 2010 and 2019 for women in cancer in all sites, lung cancer, and breast cancer. In addition, the SMRs of municipalities with the highest population were larger than other municipalities in both 2010 and 2019 for both sexes with regard to cancer in all sites.
